# Characterization of meiotic crossovers and gene conversion by whole-genome sequencing in *Saccharomyces cerevisiae*

**DOI:** 10.1186/1471-2164-10-475

**Published:** 2009-10-15

**Authors:** Ji Qi, Asela J Wijeratne, Lynn P Tomsho, Yi Hu, Stephan C Schuster, Hong Ma

**Affiliations:** 1 Center for Comparative Genomics and Bioinformatics, Pennsylvania State University, University Park, Pennsylvania 16802, USA; 2 Department of Biology, Pennsylvania State University, University Park, Pennsylvania 16802, USA; 3 The Huck Institutes of the Life Sciences, Pennsylvania State University, University Park, Pennsylvania 16802, USA; 4 The Intercollege Graduate Program in Plant Biology, Pennsylvania State University, University Park, Pennsylvania 16802, USA; 5 State Key Laboratory of Genetic Engineering, Institute of Plant Biology, Center for Evolutionary Biology, School of Life Sciences, Fudan University, Shanghai 200433, PR China; 6 Institutes of Biomedical Sciences, Fudan University, Shanghai 200032, PR China; 7 Department of Plant Cellular and Molecular Biology, the Ohio State University, Columbus OH 43210, USA

## Abstract

**Background:**

Meiotic recombination alters frequency and distribution of genetic variation, impacting genetics and evolution. In the budding yeast, DNA double strand breaks (DSBs) and D loops form either crossovers (COs) or non-crossovers (NCOs), which occur at many sites in the genome. Differences at the nucleotide level associated with COs and NCOs enable us to detect these recombination events and their distributions.

**Results:**

We used high throughput sequencing to uncover over 46 thousand single nucleotide polymorphisms (SNPs) between two budding yeast strains and investigated meiotic recombinational events. We provided a detailed analysis of CO and NCO events, including number, size range, and distribution on chromosomes. We have detected 91 COs, very close to the average number from previous genetic studies, as well as 21 NCO events and mapped the positions of these events with high resolution. We have obtained DNA sequence-level evidence for a wide range of sizes of chromosomal regions involved in CO and NCO events. We show that a large fraction of the COs are accompanied by gene conversion (GC), indicating that meiotic recombination changes allelic frequencies, in addition to redistributing existing genetic variations.

**Conclusion:**

This work is the first reported study of meiotic recombination using high throughput sequencing technologies. Our results show that high-throughput sequencing is a sensitive method to uncover at single-base resolution details of CO and NCO events, including some complex patterns, providing new clues about the mechanism of this fundamental process.

## Background

Meiosis is essential for eukaryotic sexual reproduction and reduces the number of chromosomes in half to generate haploid cells [1-3]. To ensure the proper meiotic homolog segregation, the homologs must recognize and pair with each other in early prophase I [1-3]. It is thought that a key pairing mechanism is via DNA heteroduplex formation, which is intimately coupled with the initiation of meiotic recombination [2]. One major type of outcome of meiotic recombination is crossover (CO), which involves the exchange of flanking markers, as well as possible gene conversion (GC) [4,5]. Another result of recombination is GC without exchange of flanking markers (Non-CO, or NCO) [4,5]. Meiosis is also the process that re-distributes the genetic variations in a eukaryotic population. The extent of meiotic recombination directly impacts the frequency of specific combinations of alleles. Because of the effect of meiotic recombination on the distribution of genetic diversity, meiosis is thought to have contributed to the extraordinary diversity and evolutionary success of eukaryotes [6-10].

Meiotic recombination has been studied extensively using model systems, including the budding and fission yeasts, *Drosophila melanogaster*, *Caenorhabditis elegans*, mammals, *Arabidopsis thaliana*, and maize [1-3]. In the budding yeast *Saccharomyces cerevisiae*, molecular and biochemical studies have identified key intermediates of meiotic recombination, starting with DNA double strand breaks (DSBs) and D-loops [4,5]. A portion of the D-loops proceeds to form double Holliday junctions (DHJ), which are then resolved largely to COs. Some D-loops undertake another pathway to form COs, possibly via single Holliday junctions (SHJ), as seen in the fission yeast [11]. A third option for the D-loops is the repair of DSBs without COs, resulting NCO/GC events if the two recombining DNAs are not identical.

Because recombination occurs at many sites in the genome, it is important to investigate recombination at the whole-genome level. Genome-wide genetic detection of crossovers has been done in many genetic systems, resulting in the construction of genetic maps, as well as producing other information. However, previous molecular studies usually relied on the use of naturally occurring (such as the one at the *HIS4 *locus) and artificially generated (such as ones induced by the *HO *endonuclease) recombination hotspots as substrates; therefore, the molecular details of crossovers are not available on a genome-wide level. In addition, NCO/GC has been investigated using a small number of markers or by inference at a population level. Recently, meiosis between two strains of the budding yeast has been analyzed using microarrays, providing valuable information on the frequency of CO and NCO events on a genome-wide scale [12].

As an alternative way to analyze meiotic recombination at the DNA level on a whole-genome scale, we have used the recently developed Roche GS20/FLX [13] and Illumina [14] sequencing technologies. To obtain a large number of DNA polymorphisms as markers for recombination, we used two strains of *S. cerevisiae *that have sequenced genomes: S288C and RM11-1a [15,16], which were estimated to have 0.5-1% sequence divergence distributed throughout the genome. Here we report our results from high-throughput sequencing of both the S288C and RM11-1a (hereafter referred to as RM11 for convenience) strains and four meiotic products. Over 46 thousand single nucleotide polymorphisms (SNPs) were revealed by comparison and further parsing of the two genomic sequences. Armed with these markers, we were able to detect COs, NCOs and other recombination events in meiotic products (spores) from a diploid generated by crossing S288C with RM11.

## Results and discussion

### Resequencing of the S288C and RM11 strains identified errors in reported sequences

We compared the S288C and RM11 genomic sequences and recognized 62,324 putative SNPs; however, our preliminary analysis by sequencing PCR products using the conventional dideoxynucleotide method indicated that 101 putative SNPs were actually sequencing errors in the S288C or RM11 sequences (data not shown). Therefore, we re-sequenced the S288C (12× coverage) and RM11 (15× coverage) genomic DNAs using the Illumina technology and obtained > 4.4 and 5.2 million reads, respectively (Table 1; sequence data to be submitted to Genbank). These reads covered 94% and 93% percent of the respectively public genomic sequences and provided independent verification of 46,487 SNPs (available upon request) that were previously detected by the public sequences. In addition, we found 803 and 1104 errors (Table 1) in the public S288C and RM11 sequences, respectively (available upon request), corresponding to previously identified SNPs between these sequences. Because the S288C strain is slightly different to RM11 (estimated to be 0.5-1%), the vast majority of the public sequences are identical. The sequences that agree between the two strains should be more reliable because they are supported by both sequencing projects. However, there is a very low probability that a small number of bases might be wrong. Therefore, we also compared the sequences that are in agreement between S288C and RM11 with our new data. Using our data with consistent results from at least 2 reads, we found indeed there were only a very small number of errors, 116 and 242 in the previously reported S288C and RM11 sequences, respectively, resulting in the identification of 358 new SNPs (available upon request). Our data provide strong support for over 46 thousand SNPs, which will facilitate further molecular genetic and genomic studies using these two yeast strains.

**Table 1:** Number of reads, nucleotides and genome coverage of each meiotic product and reference genome sequencing

**Template**	**# reads**	**# bps** **(Mb)**	**Average** **length (bp)**	**coverage**	**# mapped** **reads**	**% of** **reference**	**% of** **SNPs**	**# corrected** **errors**	**Substitu** **tions**	**indels**	**Sequencing** **tech**
S288C	4,446,072	155.6	35	12.9 ×	3,083,125	94%	96%	803	415	388	Illumina
RM11-1a	5,292,528	185.2	35	15.3 ×	4,119,484	93%	90%	1104	471	633	Illumina
Spore 1	344,790	55.8	162	4.6 ×	257,172	91%	90%	N/A	N/A	N/A	Roche GS20/FLX
Spore 2	340,831	59.9	176	4.9 ×	279,272	93%	92%	N/A	N/A	N/A	Roche GS20/FLX
Spore 3	298,340	56.8	191	4.7 ×	230,880	92%	90%	N/A	N/A	N/A	Roche GS20/FLX
Spore 4	416,998	44.4	107	3.6 ×	243,781	82%	80%	N/A	N/A	N/A	Roche GS20

### Sequencing of meiotic products by 454 provided a test for de novo assembly of new sequencing reads

To obtain a diploid with a large number of sequence polymorphisms, we crossed S288C with RM11; then we induced meiosis in the diploid using a standard protocol, and obtained a number of tetrads (asci) with meiotic spores (not shown). We cultured one set of four spores in a rich medium and isolated DNAs from these four cultures. These DNAs were sequenced using the 454 technology, resulting in approximately 300,000 to 416,000 reads, or 3.6× to 4.9× coverage, of each of the four meiotic products (Table 1).

Because the 454 sequences are relatively long, ranging from ~100 to > 170 bps, we thought it would be informative to assess the feasibility of performing assembly of the new sequence data as a strategy for de novo sequencing of genomes. We assembled the 454 reads from our four meiotic samples separately to test the effect of read length on assembly since the sequences from spore 4 had only shorter reads of ~100 bases, whereas the sequences of the other meiotic products had longer reads. We found that the assembly of data from any of the first three spores with longer reads gave much longer contigs (t test, p << 0.001) and a higher coverage than the short reads from spore 4, using the S288C genome as a reference (Table 2). Next, we pooled the data from two, three or four spores, and performed assembly again; we found that the data from any two of the first three spores allowed the assembly of much larger contigs and greater coverage of the genome than data from single spores (Table 2). With the combination of data of the first three spores, the assembly yielded longer contigs, but little increase in the coverage of the genome. The addition of the short reads from spore 4 primarily resulted in many more short contigs. These results indicated that ~10× coverage from the longer 454 reads provided > 94% coverage of the yeast genome.

**Table 2:** Coverage of S288C by assembled contigs based on combined reads from different spores

**Template**	**coverage**	**# contigs**	**Largest** **contig (bp)**	**Ave. contig ** **size (bp)**	**Median contig ** **size (bp)**	**N50 contig** **size (bp)**	**Assembled ** **size (Mb)**	**# assembled** **reads**	**% of** **Reference**
Spore 1	4.6 ×	12,272	8,128	768	589	1,019	9.4	302,707	79%
Spore 2	4.9 ×	9,683	9,655	1,053	779	1,505	10.2	306,022	85%
Spore 3	4.7 ×	10,281	9,586	959	721	1,323	9.9	263,286	83%
Spore 4	3.6 ×	21,513	9,081	290	254	308	6.2	242,053	40%
2 spores (1+2)	9.5 ×	3,158	42,872	3,586	2,069	7,242	11.3	644,429	94%
2 spores (1+3)	9.3 ×	3,369	34,567	3,348	1,973	6,593	11.3	601,938	94%
2 spores (2+3)	9.6 ×	2,749	38,236	4,132	2,375	8,535	11.4	594,797	94%
3 spores (1+2+3)	14.2 ×	1,836	79,085	6,235	1,560	18,824	11.4	906,455	95%
4 spores	17.8 ×	7,730	91,399	1,686	265	14,948	13.0	1,224,321	95%

Software NEWBLER (Roche off-instrument software) was used for assembly of different datasets. Contigs homologous to mitochondrial sequence of S288C were removed before further analysis.

### Analysis of SNPs in the meiotic sequence data revealed 91 COs with and without GCs

**Figure 1:**
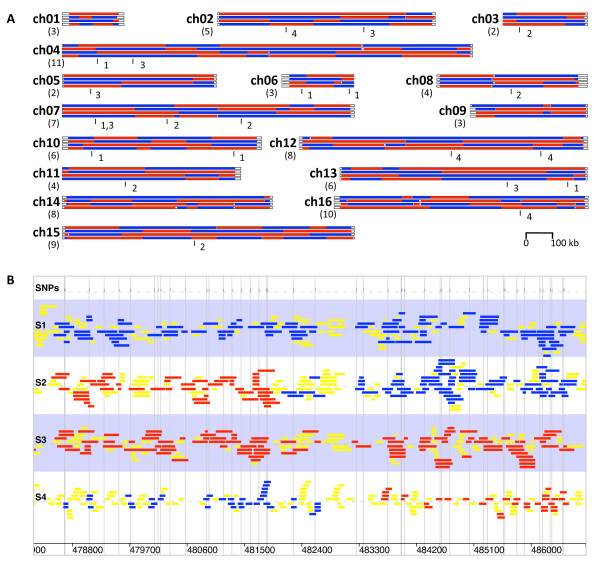
**An overview of distribution of 91 COs and 21 GCs on 16 yeast chromosomes**. (A) Sequences identical to S288C are shown as blue bars, those identical to RM11 as red bars. Empty areas near chromosome ends (repetitive telomere regions) do not have reads coverage or SNPs. There is a deduced CO event if the color changes from blue to red in one meiotic product and from red to blue in another at closely spaced positions. The number of COs on each chromosome is shown below the chromosome name. The GC positions on each chromosome are indicated by vertical lines with a number from 1 to 4 indicating the corresponding converted meiotic product. GC was not detected on chromosomes 1, 9 and 14. (B) An example of a CO from chromosome 15 shows the mapping of reads for the four meiotic products. Blue bars represent mapped reads matching the S288C sequences, red bars represent reads matching RM11. The meiotic product #1 is identical to S288C and #3 is to RM11 in this region; they were not involved in the CO event. Product #2 and #4 exchanged sequences, indicating a CO event whose minimum and maximum sizes (visible) are defined by the two horizontal range arrows at the top. Vertical gray lines show the positions of SNPs between S288C and RM11.

Using the > 46 thousand SNP markers that we have verified, we determined chromosomal regions that are primarily of the S288C or RM11 strain backgrounds, respectively (Figure 1A), resulting in the identification of 91 COs (4550 cM, close to the reported map distance of 4884 cM) (Figure 1A, Additional file 1 - Table S1). As shown in Figure 1A, each of the 16 yeast chromosomes had 2 to 11 COs, with larger chromosomes having more COs than smaller chromosomes. This observation is well supported by a recent study [12] based on 4161 COs resulted from 46 meiosis (Additional file 1 - Figure S1), in which CO number is substantially linear proportional to chromosome size (correlation coefficient squared as R^2 ^= 0.985). In addition, the genotypes of the four meiotic products indicated that the four meiotic chromatids had participated in 35, 44, 52, and 51 COs, respectively. Among the 91 CO events, 37 did not show a detectable GC (details not shown), 48 were associated with a single detected GC (see Figure 1B for an example), 5 associated with two GCs (1:3 and 2:2 [or 3:1 and 2:2] see below for more details; see Additional file 1 - Figure S2 and S3), the remaining 1 had a complex GC pattern (see below for additional information; see Additional file 1 - Figure S2 and S4). Three COs even had sequence changes in 3 of the four meiotic products. A subset of COs was verified by PCR and the results (Additional file 1 - Figure S5) agreed with the high-throughput sequencing results.

### Comparison of meiotic sequences uncovered 21 NCOs/GCs

In addition, by comparing sequences from all four meiotic products, we detected 21 putative GC events not associated with CO (Additional file 1 - Table S2). To verify its reliability, we analyzed the DNA sequences at all 21 putative GC sites using PCR and conventional Sanger DNA sequencing. The PCR and sequencing results were in complete agreement with the Roche GS20/FLX and Illumina results. The results indicated that the four meiotic chromatids had 7, 6, 4, and 4 detected GCs. Because the two yeast genomes are ~99% identical, the observed GC events were likely fewer than the actual recombination/pairing events. We estimated the possible number of undetected NCOs in a way similar to that in a recent study [12]. Among 91 COs discovered in this analysis, 37 were detected using flanking SNP information, but did not show a detectable GC due to the lack of a SNP. If a similar fraction (37/91 = 0.407) of NCOs was not detected due to the lack of SNPs, the estimated total amount of NCOs would be 30 (= 21 × 1.407). Therefore, our genome sequencing results indicated that there were a significant number of NCO (GC) events, resulting in a change of allelic frequency.

The DNA of spore 4 was analyzed earlier than others and the 454 reads had shorter lengths, resulting in a reduced coverage of the SNPs. One effect of the reduced coverage was that a crossover involving spore 4 probably had more inaccurate border(s); nevertheless, all COs involving spore 4 were still detectable because flanking markers were still observed. Because NCOs were detected using the SNP information for each spore in the chromosomal context, reduced SNP coverage in spore 4 likely caused a decrease in NCO detection, providing another possible explanation for under-estimation of the NCO number.

### Size range and map position of COs and NCOs

**Figure 2:**
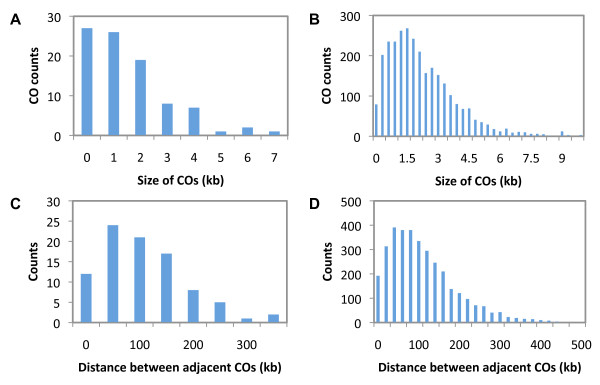
**Distribution of CO size and distance between adjacent COs**. (A) Median sizes of 91 COs; (B) Sizes of 4161 COs detected using 46 meiosis from a recent study [12]; (C) Distribution of distances of adjacent COs among the detected 91 COs; (D) Distribution of distances of adjacent COs among the reported 4161 COs [12].

From the sequence information, we estimated the minimum and maximum sizes of the COs, as illustrated in the example shown in Figure 1B. The maximum possible lengths of crossover regions (defined by the closest detected markers) ranged from 164 bp to 10,637 bp. As this could be an over-estimation due to the limited SNP information, we also estimated the minimum size, as defined by the detected SNPs within the CO regions; as often there was only one SNP in the CO region, the minimum sizes for these were as small as one base pair. Therefore, median sizes of COs, estimated by the average value of their minimum and maximum sizes, were used for statistical analysis (Figure 2A). A histogram for over 4000 COs detected from 46 meiosis from a recent study [12] is shown in Figure 2B as a comparison (1252 COs without available length were not included). Distribution of distance between adjacent COs are displayed in Figure 2C and 2D for both 91 COs in this analysis and that from the recent study [12].

Nevertheless, at least 28 COs had minimal sizes of greater than 1.0 kb, with the largest minimum size being over 7 kb (Additional file 1 - Figure S6). Among the NCOs, the maximum sizes ranged from 1,109 bp to 7,575 bp, and the largest minimum size was over 6.5 kb (Additional file 1 - Figure S7). These results indicate that both CO and NCO can involve several kbs, suggesting that DNA repair and/or heteroduplex formation can be rather extensive. In budding yeast, most COs are thought to result from the double Holliday junctions (DHJs), and a small fraction of COs from single Holliday junctions (SHJs) [17]. If all DHJ are initiated with the same size and then each Holliday junction "randomly" expands to a larger size, the length distribution of COs should follow a Normal distribution. However, we found that the observed sizes of COs (Figure 2A) were not consistent with a Normal distribution, supporting a mixture of COs resulted from both DHJs and SHJs, since COs from SHJs might have different ranges of lengths resulted from a different pathway [18]. This distribution is also supported by the same analysis on the data of the recent study using microarrays (Figure 2B) [12].

**Figure 3:**
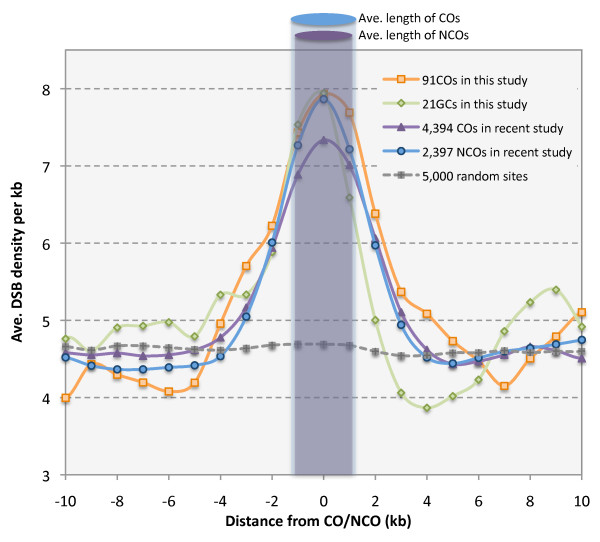
**Close correspondence of CO/NCO loci to hotspots for double stranded breaks (DSBs)**. We examined DSB densities in 20-kb regions flanking each locus in different sets of COs/NCOs from our studies and those reported by Mancera et al. [12]; Genome-wide DSB density data from 40,000 probes were obtained from Buhler et al. [19], and average densities for 1-kb intervals (centered at the position shown on the X-axis) in 10-kb regions upstream and downstream regions flanking each locus in a specific set of COs or NCOs. The yellow (square) and green (diamond) lines are for the 91 COs and 21 GCs from this study, respectively, whereas the purple (triangle) and blue (circle) lines are for the 4,394 COs and 2,397 NCOs, respectively, in the Mancera et al. study [12]. All four datasets show a clear correspondence of CO/NCO sites to the highest average DSB densities, suggesting that they are enriched for DSB hotspots. The grey line represents the DSB densities of 20-kb regions around 5,000 random sites, as a negative control. The two ovals at the top indicate the estimated average length of COs/NCOs from our study.

A recent study mapped 1,306 DSB hot-spots along the chromosomes of a *dmc1 *mutant [19]. To test whether the CO and NCO events were enriched for DSBs, we obtained the DSB density data [19] in a region from 10-kb upstream to 10-kb downstream of each CO, and calculated the average DSB density in 1-kb intervals centered at every kb in this 20-kb region for the CO and NCO sets in our study, and found that the peak of highest average DSB density was very close to the site of CO or NCO (Figure 3). We also performed a similar analysis for the CO and NCO loci reported by Mancera et al. [12], and found very similar patterns (Figure 3). These findings indicated that the loci of COs and NCOs were enriched for DSB hot-spots, consistent with the idea that DSBs are initiation sites of meiotic recombination.

**Figure 4:**
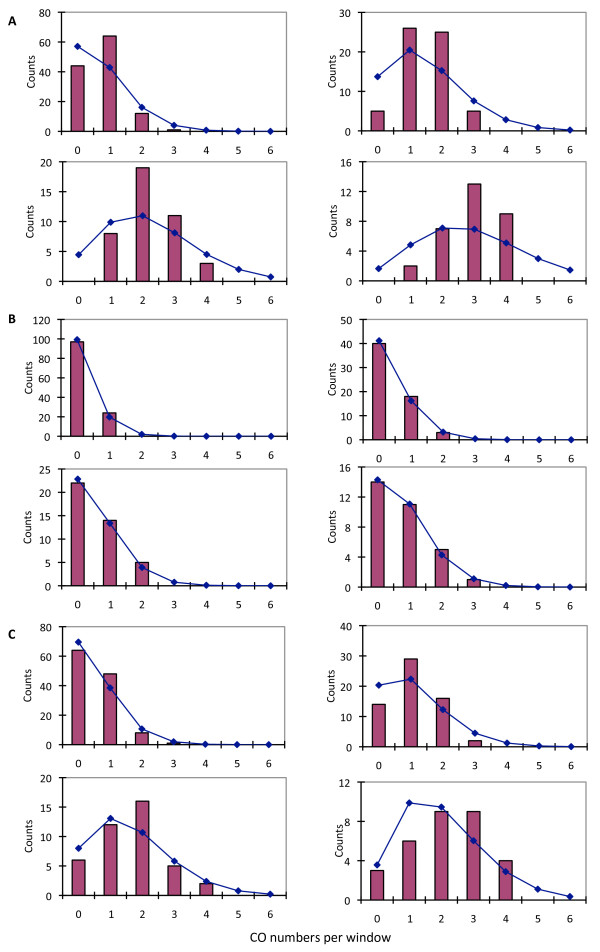
**Distribution of COs on the genome comparing with Poisson model**. (A) the distribution of all 91 COs suggests COs interference. Four different bin sizes are used, 100 kb for left upper graph, 200 kb for right upper one, 300 kb for left lower one, 400 kb for right lower one; (B) the distribution of small COs (max size < 1500 bp) is consistent with Poisson model; (C) the distribution of large COs (max size > 1500 bp) shows deviation from Poisson model, suggesting COs interference.

In yeast, genetic studies indicated that COs might be distributed according to two models: Poisson and counting models for interference insensitive and sensitive COs, respectively [18,20]. We found that the observed COs (Figure 4A) did not agree with a Poisson model. Further analysis indicates that the distribution of COs is consistent with a mixture of interference insensitive and sensitive events (Figure 4B and 4C). In the budding yeast, plants, and human, interference-sensitive COs are the majority, accounting for about 80% of the total CO events, with the remaining COs being interference insensitive [18,20-23]. Molecular genetic studies indicate that interference-sensitive COs are generated from the DHJ intermediates, and involve branch migration, resulting in potentially longer tracks of conversion [24]. In the fission yeast, the interference insensitive pathway has been reported to go through a SHJ intermediate, having a distinct mechanism [11], although the mechanisms for interference insensitive COs in other organisms are not clear.

The maximum possible lengths of CO regions in this study covered a wide range. If interference insensitive COs in the budding yeast also involve a SHJ, it is possible that shorter COs might be generated by the interference insensitive pathway. To test this idea, we analyzed the genomic distribution of the COs that were shorter than 1.5 kb, and found them to be consistent with a Poisson distribution (Figure 4B); on the other hand, the COs that were longer than 1.5 kb did not have a Poisson distribution, consistent with the possibility that they were generated by the interference-sensitive pathway (Figure 4C). Analyses with different cutoffs other than 1.5 kb were also preformed (data now shown), but the statistical fit of the distribution of shorter COs to a Poisson model was not as good as that of the 1.5 kb cutoff; in addition, the proportion of shorter COs from the 1.5 kb cutoff was consistent with previous observations [18,20-23].

**Figure 5:**
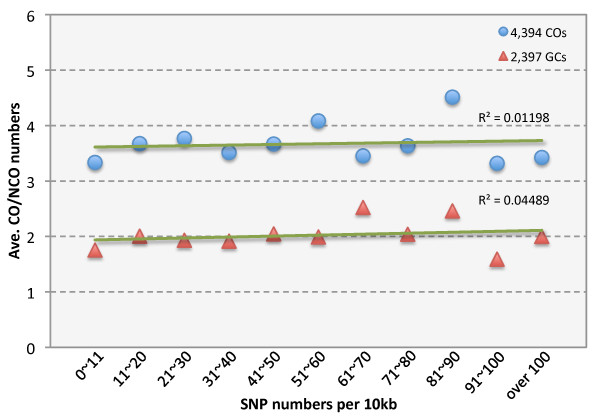
**Evenly distributed COs/NCOs among regions of different SNP density**. We examined CO/NCO frequencies in regions of different SNP densities with 10-kb window size and found the numbers of loci of COs/NCOs in a recent study were not correlated with SNP densities. The blue and red dots are for 4,394 COs and 2,397 NCOs, respectively, reported in Mancera et al. [12]. The lack of significant correlation is indicated by the nearly horizontal green lines, with R square values of 0.01 and 0.04 for COs and NCOs, respectively. The slightly higher value for NCOs might suggest that low-SNP density regions could have a higher failure rate in detection of NCOs.

Grieg et al. [25] reported that sequence divergence between homologs could affect the frequency and distribution of COs. To test whether the sequence differences between S288C and RM11 had an effect on CO and NCO frequency, we divided the yeast genome into 10-kb intervals, and determined the distribution of numbers of SNPs in 10-kb intervals throughout the genome. We then obtained the positions of the COs and NCOs from Mancera et al. [12], and plotted the average number of COs (or NCOs) as a function of the number of SNPs/10-kb (Figure 5). Our analysis showed that the regions with more SNPs did not have a reduced frequency of COs or NCOs. Therefore, the extent of divergence between the S288C and RM11 strains did not seem to adversely affect CO frequencies.

### Sequence data revealed complex CO and post-meiotic segregation events

**Figure 6:**
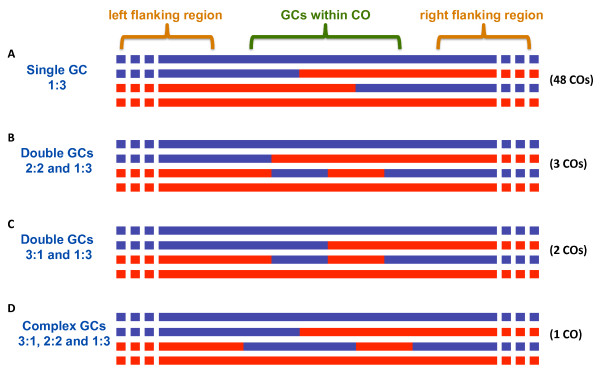
**A summary of different patterns of GCs within COs**. (A) "Single GCs" (1:3 or 3:1) were detected in 48 COs. Only the 1:3 GC pattern is shown here as an example; (B) three COs were associated with "Double GCs" (2:2 and 1:3) whose mapping details were shown in Additional file 1 - Figure S2; (C) two COs contained "Double GCs" with pattern as 3:1 and 1:3 whose details were shown in Additional file 1 - Figure S2D and E; (D) A complex pattern, 3:1, 2:2 and 1:3, was detected in one CO, see Additional file 1 - Figure S3.

As mentioned above, several COs have complex GC patterns (Figure 6 and Additional file 1 - Figure S2, S4 and S8), consistent with the repair of heteroduplex DNA after Holliday junction formation and resolution. In addition, we also found that three CO events had sequence alterations in three of the four meiotic products (Additional file 1 - Figure S5) [26,27]. Further analysis of these three events using PCR and sequencing showed that they indeed involved three chromatids (Additional file 1 - Figure S5), suggesting that DSBs were generated in two chromatids, and that the recombinogenic broken ends from the DSBs interacted with the other two chromatids during the recombination process, in four-chromatid events. In addition, we observed one region that had two adjacent GC regions without exchange of flanking sequences (Additional file 1 - Figure S9); this could most easily be explained by the resolution of a DHJ in a NCO fashion [26,27]. Although, this was proposed in the original DSB repair model of recombination as a major pathway for NCOs, more recent models favor a non-Holliday junction pathway for NCOs. Our results suggested that DHJs might still be revolved to form NCOs, although at a frequency much lower than that of CO formation. We have also detected some evidence of post-meiotic segregation (PMS), which was an indication of unrepaired heteroduplexes that subsequently segregated during mitotic growth of the haploid meiotic products (Additional file 1 - Figure S10). From the high-throughput reads, we found one putative PMS events and another 4 PMS candidates with low quality from initial mapping. Sequence analysis of PCR products confirmed the PMS event and the other candidates were denied due to mis-alignment of repeat sequences.

A major difference between this study and the microarray studies published recently [12] is that we determined the actual sequences of the meiotic products, rather than inferring about the SNP genotypes on the basis of differential hybridization signals. Our approach can detect both SNPs and any other sequence information. It was reported that spontaneous mutation rates at specific loci could be 6-20 fold higher in meiosis than mitosis [28,29]. However, there has been no study of mutations during meiosis at a genome-wide scale. To search for spontaneous mutations, we examined the sequences throughout the genome for base substitution mutations and did not identify any sequences that differed from both parental sequences. Therefore, the mutation rate was below our detection limit of ~8 × 10^-8 ^per base per cell division. A recent genome-wide analysis of mitotic yeast cells provided an estimated rate of mitotic substitution of 3.3 × 10^-10 ^per base per cell division [30], suggesting that a 6-20 fold increase would not be detected by our analysis. Tandem repetitive sequences are known to have high mutation rates to form different copy numbers in cell division. Repeats with a higher copy number usually have higher mutation rates and lower appearance frequency (number of loci) [30]. However, the possibility of appearance of such kind of mutation is still too low to be observed in one generation of meiosis, as confirmed by our analysis of all 16 chromosomes in the 4 spores.

## Conclusion

In summary, our studies have reliably verified over 46 thousand SNPs that were identified by comparison between the public S288C and RM11 genomic sequences and have uncovered errors in the S288C and RM11 sequences, respectively, thereby removing 1907 previously reported SNPs and defining 358 new SNPs. These new sequence results are useful resources for further genomic and genetic studies using the budding yeast. We have uncovered detailed molecular information about meiotic recombination on a whole genome level using high-throughput sequencing. The numbers of CO and NCO events we detected were in very good agreement with previous studies; furthermore, we described complex patterns of COs that involved three chromatids, shedding new light on the process of meiotic recombination. Our studies provide a window into the nature of meiotic recombination at the DNA level throughout the genome and established a whole-genome foundation for further molecular genetic studies of this fundamental process.

## Methods

### Growth of yeast cells

The *Saccharomyces cerevisiae *strains S288C and RM11 were grown overnight at 30° on an agar plate with the YPD rich medium, and mixed on an YPD plate to allow mating to form diploid cells. Newly formed zygotes were identified under a light microscope and transferred to a clean area of the YPD plate using a micromanipulator, and grown to a colony at 30°. The diploid strain was then grown on an YPD plate as a patch, and freshly grown cells were transferred to a sporulation plate. After one week, tetrads with four spores were detected under a light microscope, were partially digested in an aqueous solution of zymolyase. The partially digested tetrads were dissected to separate the spores under a light microscope using a micromanipulator, and the spores were allowed to grow for two days on an YPD plate into colonies. Cells from the colonies were used to inoculate liquid YPD cultures. Also, S288C and RM11 were similarly grown in YPD cultures to late exponential phase. The yeast cells were then harvested from the cultures and used for the isolation of genomic DNAs.

### Genomic sequence data sets

The public whole genome sequences of the S288C and RM11 strains were downloaded from NCBI (National Center for Biotechnology Information, ) and Broad Institute  respectively. The four haploid meiotic products from the same meiosis were sequenced by using Roche GS20/FLX pyrosequencing technology to detect COs, NCOs and other recombination events. The S288C and RM11-1a genomic DNAs were sent to Fasteris  for re-sequencing by using Illumina sequencing technology to verify SNPs between these two parental references. The public S288C and RM11-1a genomic sequences were used for BLAST analysis to map the newly obtained sequences from the high throughput shotgun sequencing technologies.

### Reads Mapping and SNPs Detection and Correction

We applied a series of steps to map the high-throughput reads to the S288C and RM11-1a public sequences and to detect SNPs.

First, SNPs between S288C and RM11-1a were initially identified by the global alignment tool MUMMER [31]. Ambiguous differences in repetitive and low complexity regions were ignored (the option "--mum" was used for anchoring matches uniquely on both references genomes). Total 62,324 SNPs were detected for all 16 pairs of chromosomes.

However, some SNPs were false positive and could be attributable to the sequencing error on either S288C or RM11. Each sequencing error on reference genomes could raise an artifact of gene conversion. In order to identify and then exclude these pseudo-SNPs from our analysis, S288C and RM11 were re-sequenced by using Illumina sequencing technology. 803 and 1104 nucleotides on the public S288C and RM11 were corrected by mapping of their re-sequenced reads. 46,487 of 62,324 SNPs were verified for further analysis. A confirmed SNP in this analysis must have at least 2 Illumina reads from each of S288C and RM11. Those SNPs without coverage by Illumina reads on either S288C or RM11, due to uneven sequencing coverage or matches to repeats, were removed in the analysis. These filtered out SNPs need to be verified by additional sequencing coverage.

Third, the reads from the four meiotic products were mapped to the pubic S288C and RM11 sequences by BLASTN [32] to provide primary information of location and identity for further alignment. A global identity cutoff of 80% was applied to all read matches, from which reads with high identity to reference genomes were kept. Then nucleotide sequences of the references near each SNP and the reads of meiotic products nearby were selected for detailed multiple alignment by CLUSTALW [33].

Last, whole genome mapping and visualization were applied to all 4 meiotic products near the SNPs. We developed a whole genome visualization tool, named inGAP to display all homology exchange among meiotic products. The manuscript has been submitted (Ji Qi, Fangqing Zhao, Anne Buboltz and Stephan C. Schuster) and the software is available online at 

We have also written an additional set of scripts to perform the bioinformatic analyses in this study. More information will be provided if requested.

## Authors' contributions

JQ carried out the bioinformatics analysis; HM, AJW and YH performed the cell culture, DNA preparation and PCR experiments; LPT prepared DNA library and conducted Roche/454 sequencing; HM and JQ carried out the genomic studies and drafted the manuscript; HM, SCS and JQ produced the final version of the manuscript; HM and SCS designed the study. All authors read and approved the final manuscript.

## Supplementary Material

Additional file 1 **Supplemental figures and tables**. 10 supplemental figures are displayed for selected COs and GCs with PCR results. The positions of all 91 COs and 21 GCs are listed in two tables respectively. Click here for file
