# Supplementary material for: Characterization of meiotic crossovers and gene conversion by whole-genome sequencing in Saccharomyces cerevisiae
Source: BMC Genomics. 2009 Oct 15;10:475. doi: 10.1186/1471-2164-10-475 (PMC2770529; doi:10.1186/1471-2164-10-475)
Supplement: Additional file 1 — Supplemental figures and tables. 10 supplemental figures are displayed for selected COs and GCs with PCR results. The positions of all 91 COs and 21 GCs are listed in two tables respectively. [file 1471-2164-10-475-S1.DOC]

Supplementary for “Characterization of meiotic crossovers and gene conversion by whole-genome sequencing in *Saccharomyces cerevisiae*”

Contents

1. Supplemental figures

Figure S1: Correlation of CO number and chromosome size

Figure S2: Display of different CO results after break of double holiday junctions

Figure S3: Display of COs which associate with double GCs

Figure S4: Display of COs which associate with complex GCs

Figure S5: Display of COs that involved multiple chromotids

Figure S6: The longest cross-over

Figure S7: The longest gene coversion

Figure S8: An example of unrepaired heteroduplexes between a telomere ends and a CO

Figure S9: A region has two adjacent GC regions without exchange of flanking sequences

Figure S10: A CO with a post-meiotic segregation (PMS) event

1. Supplemental tables

Table S1: List of all 91 COs for all 16 chromosomes

Table S2: List of all 21 GCs for all 16 chromosomes

Part 1. Supplemental figures


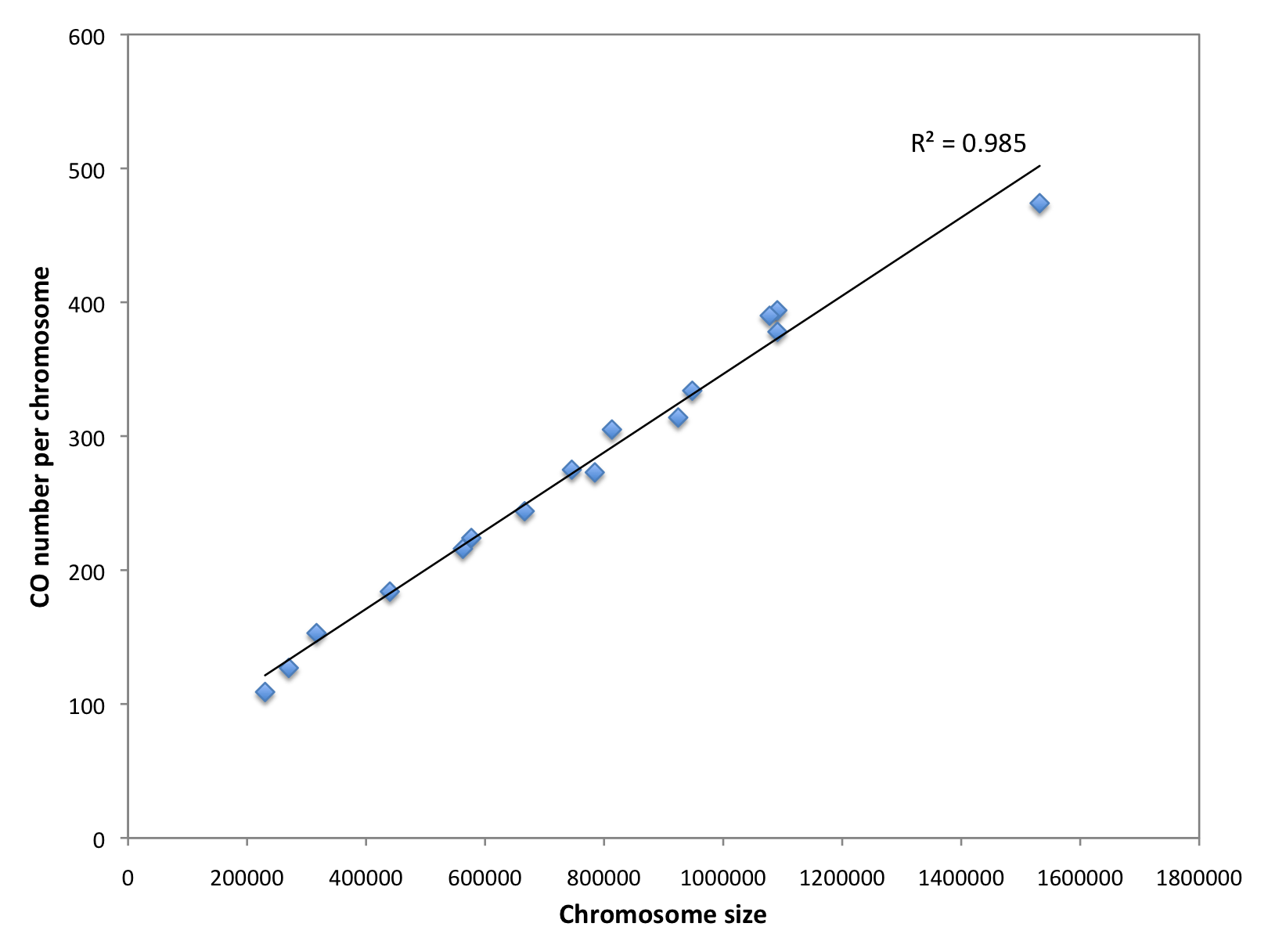


**Figure S1. Correlation between CO number and chromosome size.**

A linear relationship between CO number per chromosome and chromosome size is well supported by counting 4161 COs from 46 meiosis (Steinmetz’s data) with correlation coefficient squared as R^2^=0.985.


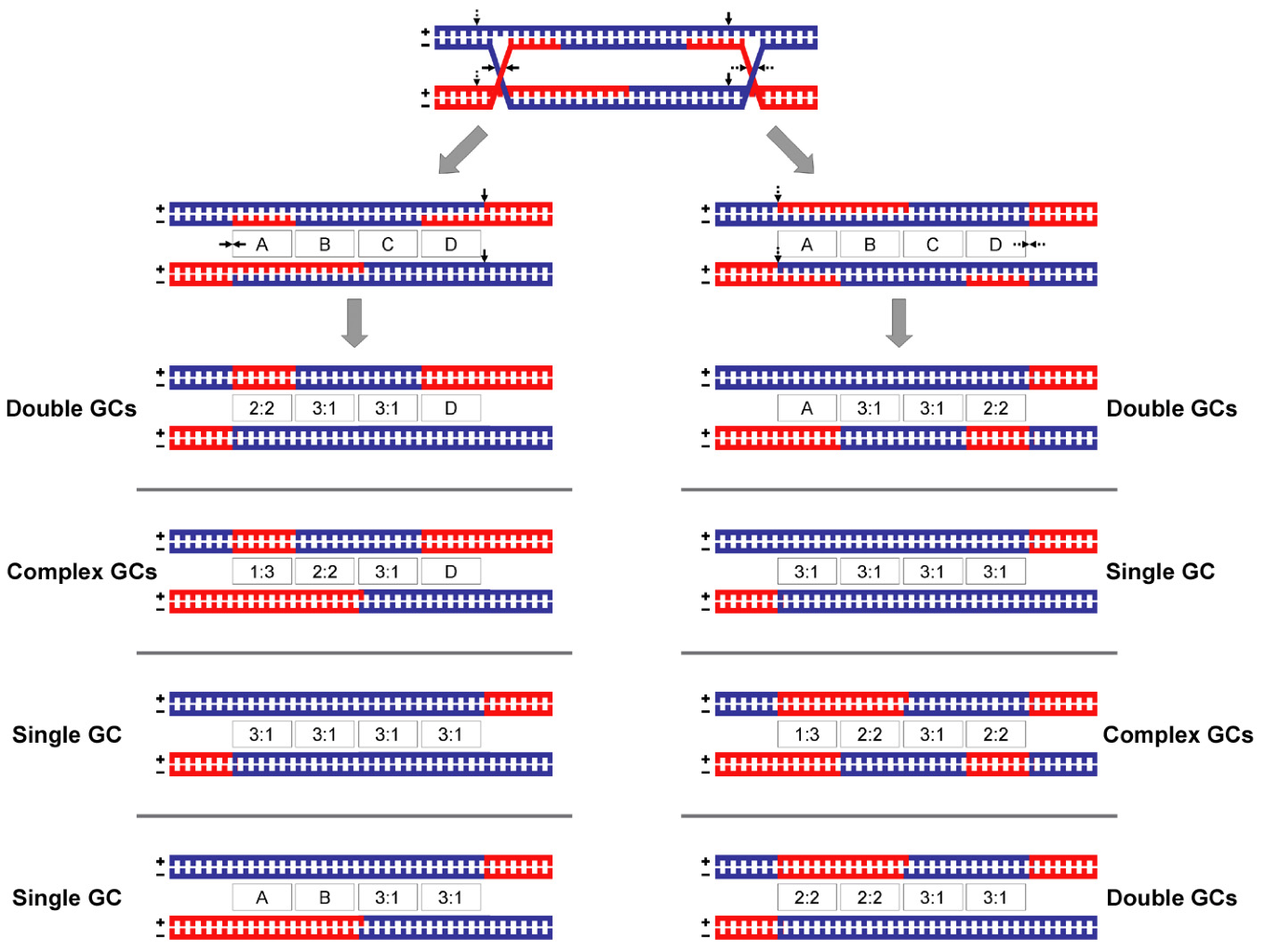


**Figure S2. Different CO possible results after resolution of double holiday junctions.**

Nicks in the same strands (horizontal arrows) for left junctions and nicks in the other strands (vertical arrows) for right junctions lead to the left type, otherwise to the right type. Each type could give four different results due to different DNA repairing outcomes (which strand is used as the “correct” one). Among the resulting 8 types, 3 COs only contain 1:3 (or 3:1) GCs and are denoted as “Single GC”; 3 COs containing one 1:3 GC (or 3:1 GC) and 2:2 GC as “Double GCs”; the remaining 2 CO types as “Complex GCs”. We note that a “Complex GC” could be seen as a “Double GC” due to insufficiency of sequence divergence and a “Double GC” could also appear as a “Single GC” due to a lack of SNPs.


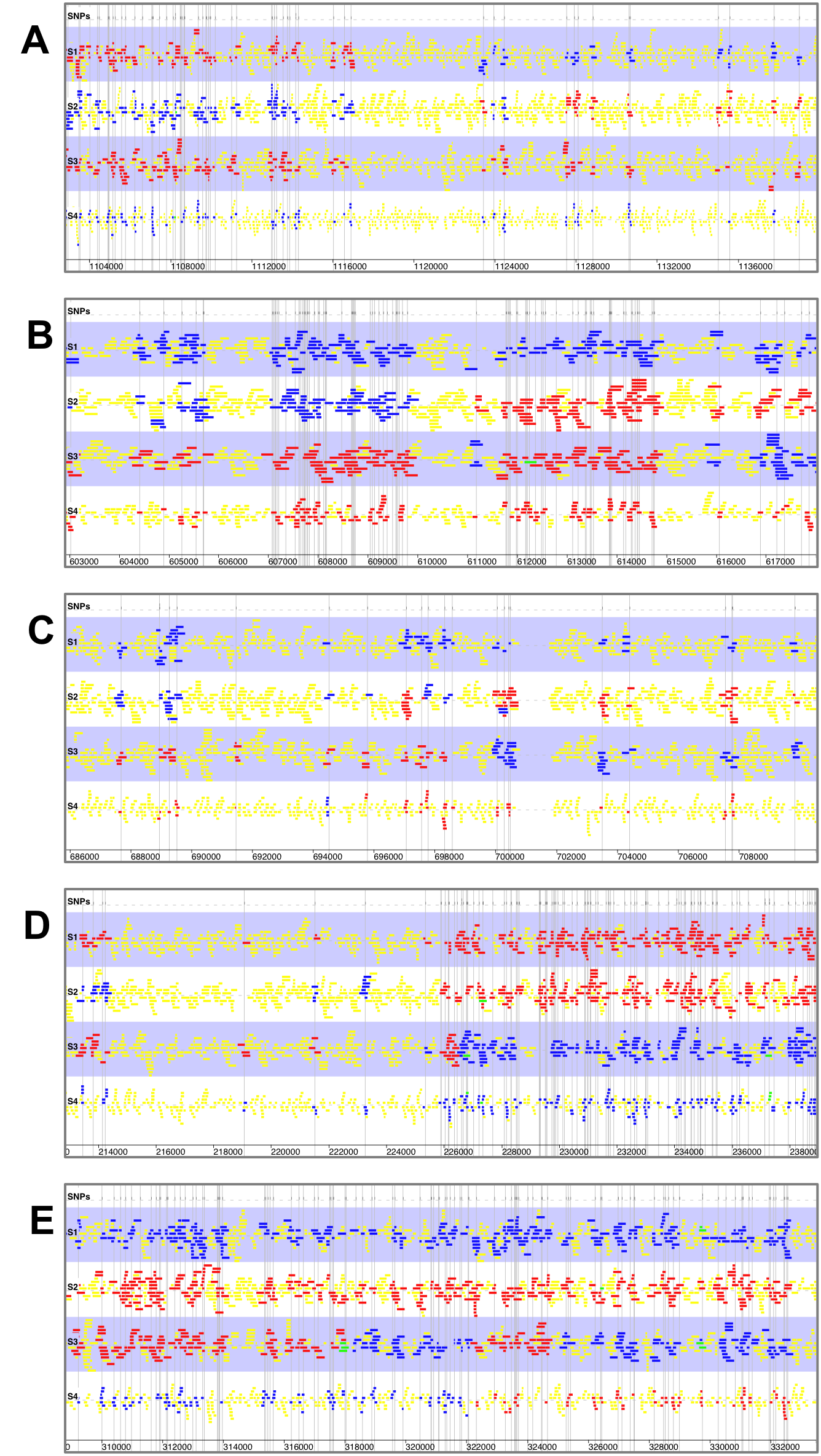


**Figure S3. Display of COs which associate with double GCs.**

(A) A CO with the meiotic chromatids #1 and #2 to form a 2:2 GC and a 3:1 GC (chromosome 4: 1,116,902~1,127,539bp). (B) A CO with the meiotic chromatids 2 and 3 to form a 2:2 GC and a 1:3 GC (chromosome 12: 609,793~616,058bp). (C) A CO with the meiotic chromatids 2 and 3 to form a 1:3 GC and a 2:2 GC (chromosome 16: 695,784~700,040bp). (D) A CO with the meiotic chromaids 2 and 3 to form a 1:3 GC and a 3:1 GC (chromosome 6: 221,516~226,608bp). (E) A CO with the meiotic chromatids 3 and 4 to form a 3:1 GC and a 1:3 GC (chromosome 12: 317,990~325,272bp).


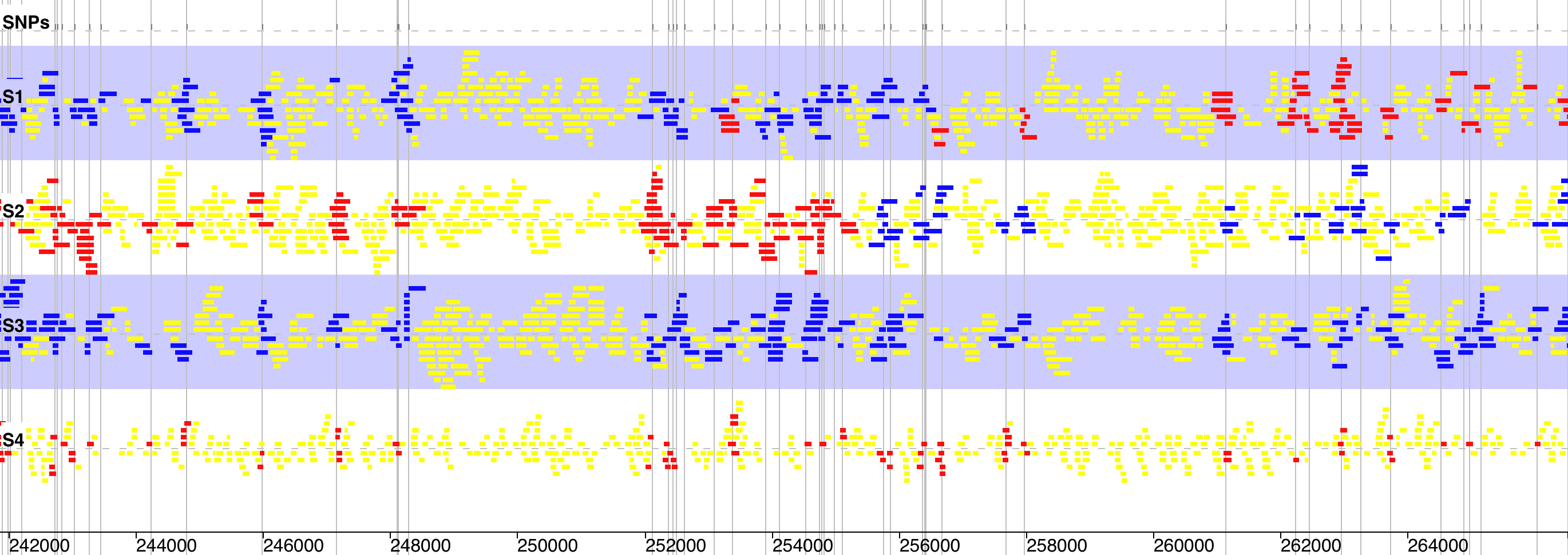


**Figure S4. One case of COs with meiotic chromatids 1 and 2 with complex GCs is shown and a 1:3 GC, a 2:2 GC and a 3:1 GC inside (chromosome 16: 253,089~256,671bp).**


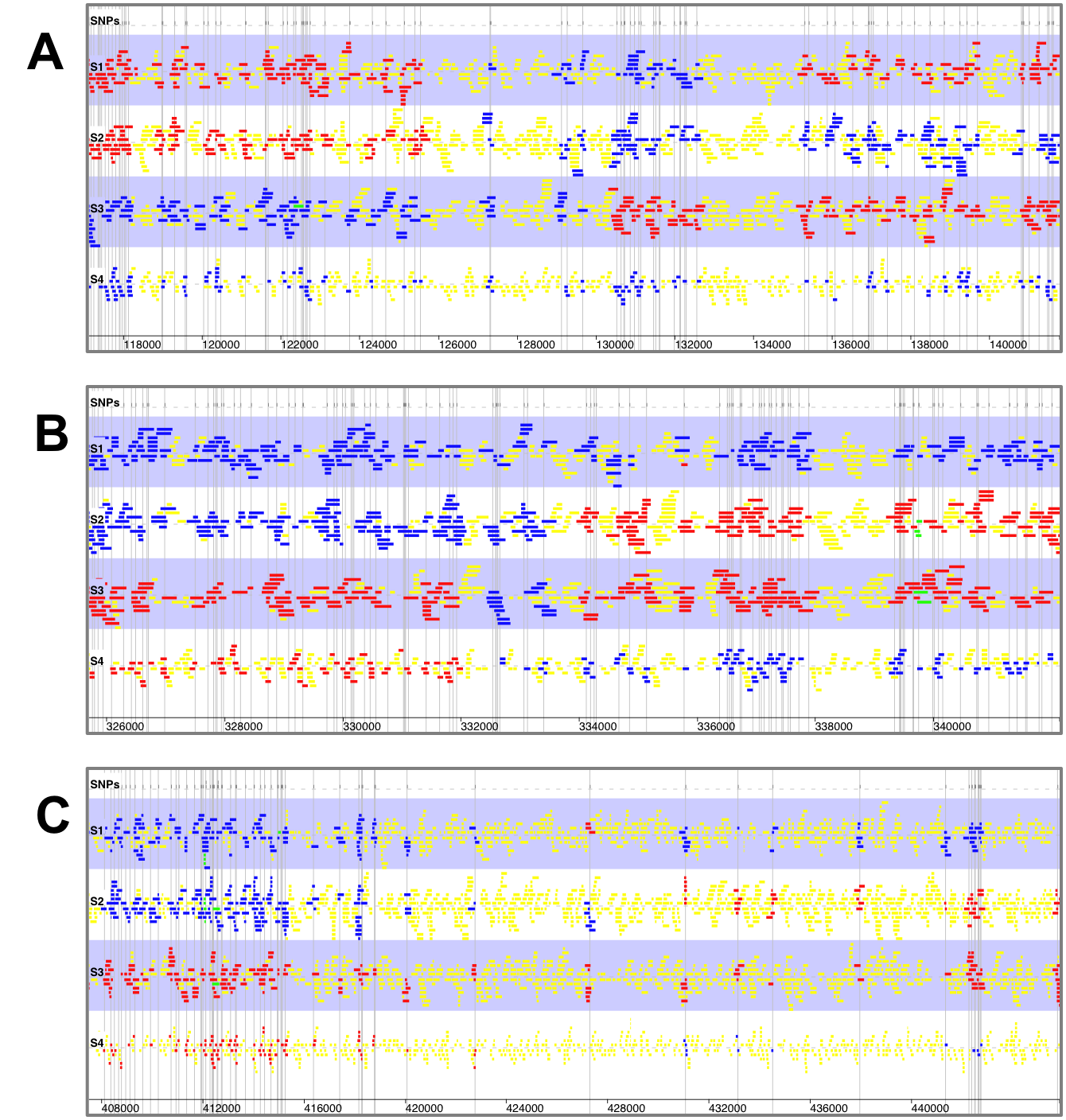


**Figure S5. Three COs with the multiple chromotids being involved.**

(A) A CO that exchanged sequences of the meiotic chromatids 2 and 3 and that converted chromatid 1 sequences (chromosome 4: 125,548~130,518bp). Details of PCR and sequencing for product 1, S288C and RM11 are displayed in the lower graph. (B) A CO that exchanged sequences of the meiotic chromatids 2 and 4 and converted that of chromatid 3 (chromosome 10: 331,929~334,120bp). (C) A CO with exchange of sequences of the meiotic chromatids 2 and 4 and conversion of that of chromatid 1 (chromosome 14: 422,748~431,065bp).


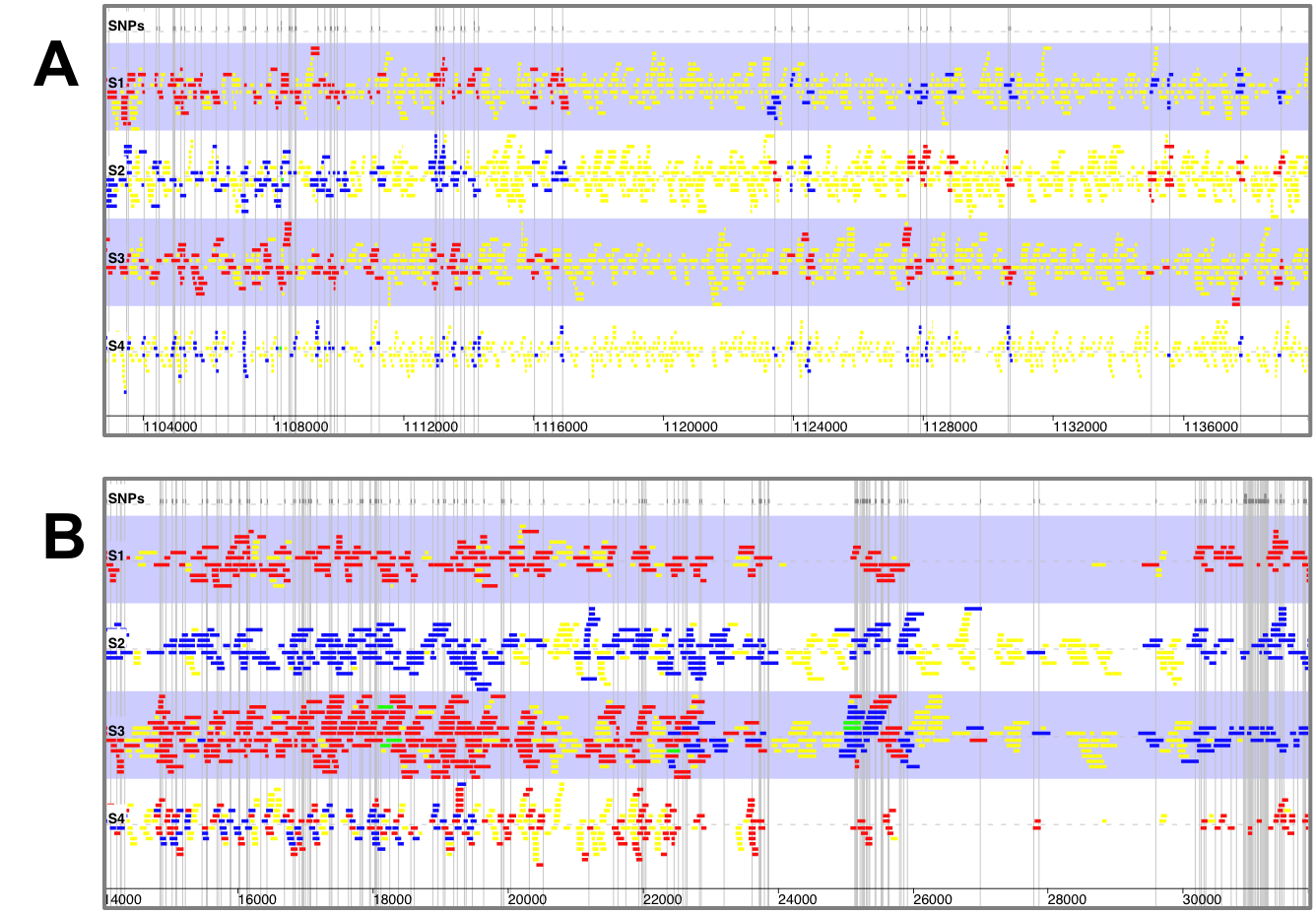


**Figure S6. Display of two longest COs .**

(A) Display of the longest CO (according to COs’ maximum size, 10,637bp) with the meiotic chromatids 1 and 2 (chromosome 4: 1,116,902~1,127,539bp). (B). Display of the longest CO (according to COs’ minimum size, 8,322bp) with the meiotic chromatids 3 and 4 (chromosome 15: 19,470~27,792bp).


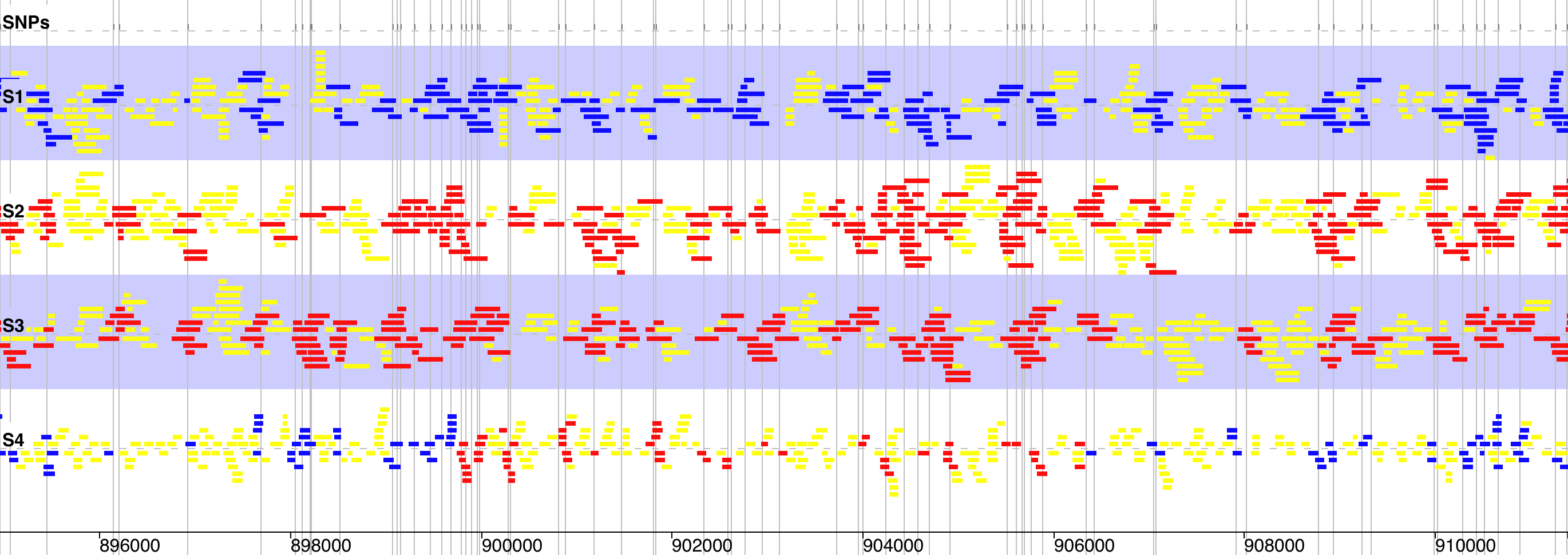


**Figure S7. Display of the longest GC (according to GCs’ minimum size, 6542bp) with the conversion of meiotic chromatid 4 (chromosome 12: 899,794~906,336bp).**


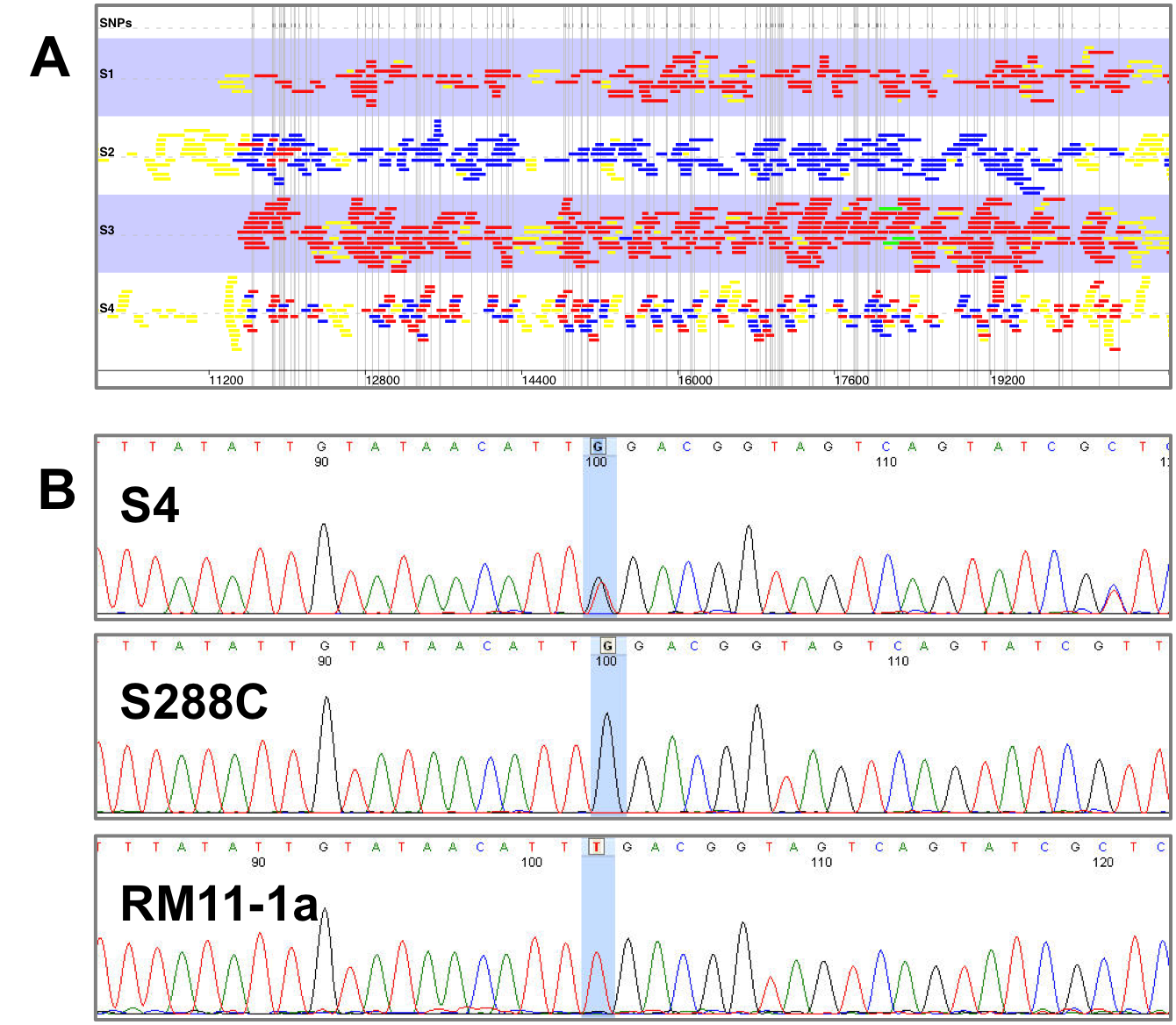


**Figure S8. A 10kb region with unrepaired heteroduplexes and PCR results.**

(A) Display of reads mapping between a telomere ends (around 11,647bp) and a CO (around 19,470bp). (B) The PCR and sequencing results show nucleotide of “G” and “T” on S288C and RM11 respectively and a mixture of “G” and “T” on the meiotic product 4.


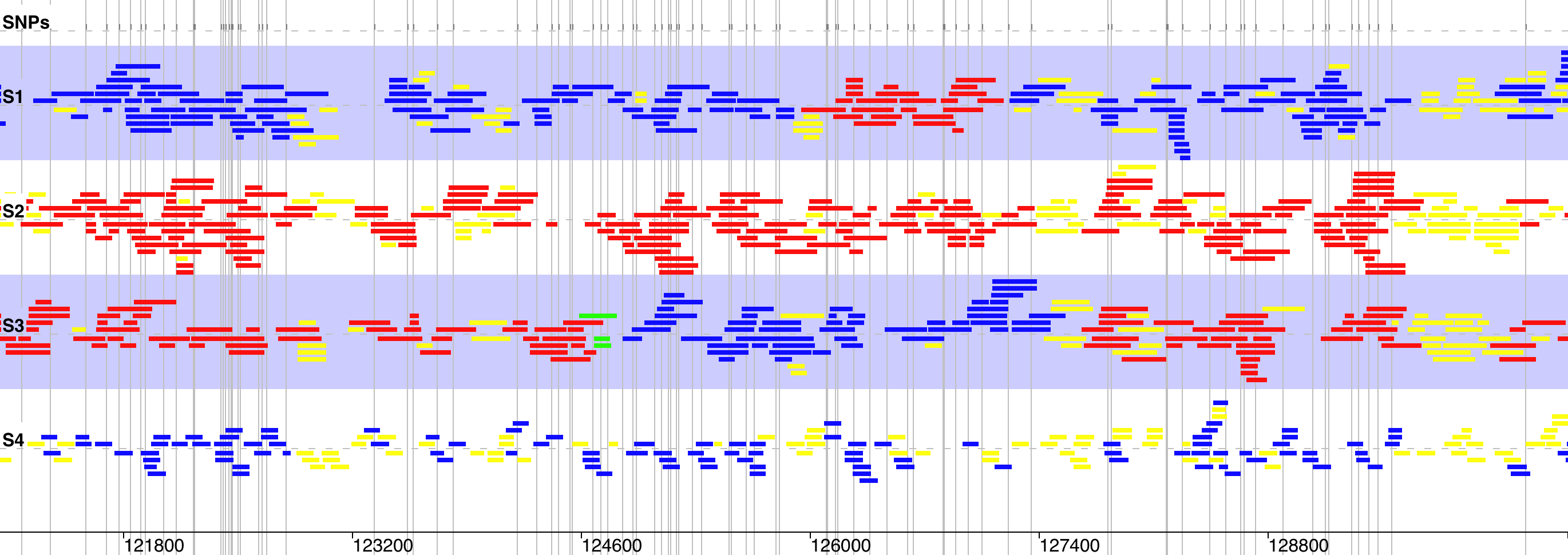


**Figure S9. A region on chromosome 7 has two adjacent GC regions without exchange of flanking sequences; this suggests that double-Holliday junctions might still be revolved to form NCOs.**


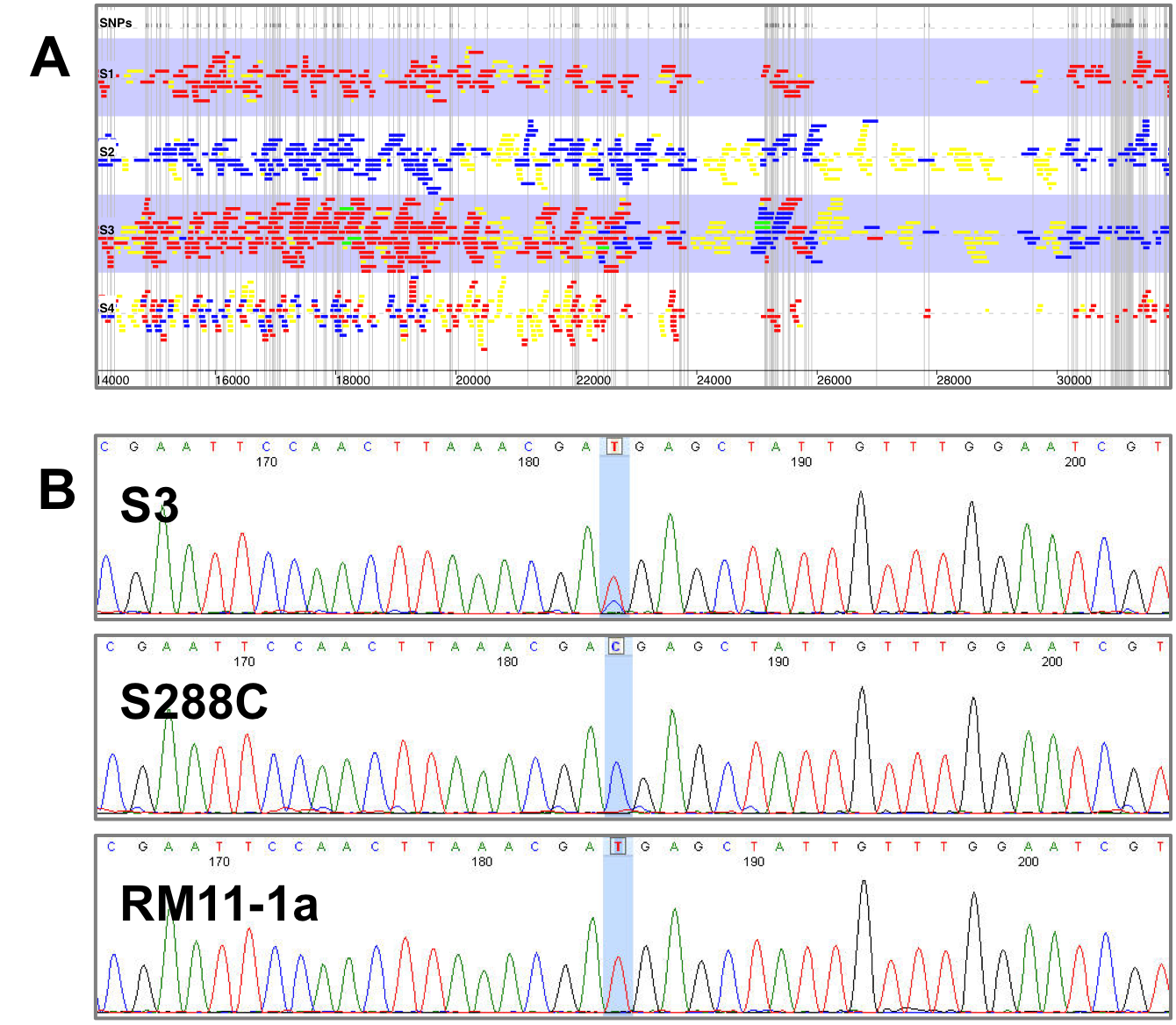


**Figure S10. A CO with a post-meiotic segregation (PMS) event and PCR results.**

(A) Display of reads mapping of this region (chromosome 15: 22,457~27,792bp). (B) Displays of the proof from PCR products around position 22,623bp. PCR products for S288C and RM11 showed nucleotides at this position as “C” and “T” respectively; the sequence for meiotic chromatid 3 was a mixture of “C” and “T”.

Part 2. Supplemental tables

Table S1. List of all 91 COs for all 16 chromosomes

| Chromosome | Start position | End position | Involved products |
| --- | --- | --- | --- |
| ch01 | 64484 | 65432 | 2, 4 |
| ch01 | 109736 | 116328 | 2, 4 |
| ch01 | 191698 | 192334 | 3, 4 |
| ch02 | 196420 | 196584 | 3, 4 |
| ch02 | 359976 | 360860 | 3, 4 |
| ch02 | 458913 | 460196 | 2, 3 |
| ch02 | 639422 | 644140 | 2, 4 |
| ch02 | 700731 | 705101 | 1, 2 |
| ch03 | 51650 | 53891 | 1, 3 |
| ch03 | 235557 | 239953 | 2, 4 |
| ch04 | 60461 | 63840 | 1, 4 |
| ch04 | 125548 | 130518 | 2, 3 |
| ch04 | 386142 | 388144 | 1, 2 |
| ch04 | 505249 | 505545 | 2, 4 |
| ch04 | 588709 | 589438 | 1, 3 |
| ch04 | 687629 | 694030 | 3, 4 |
| ch04 | 770349 | 774846 | 3, 4 |
| ch04 | 1019306 | 1022767 | 3, 4 |
| ch04 | 1116902 | 1127539 | 1, 2 |
| ch04 | 1267942 | 1270025 | 2, 4 |
| ch04 | 1474562 | 1474877 | 2, 3 |
| ch05 | 222252 | 222508 | 3, 4 |
| ch05 | 489422 | 492124 | 2, 3 |
| ch06 | 92117 | 94475 | 1, 3 |
| ch06 | 196304 | 197390 | 3, 4 |
| ch06 | 221516 | 226608 | 2, 3 |
| ch07 | 272231 | 273430 | 2, 4 |
| ch07 | 418506 | 420888 | 1, 4 |
| ch07 | 436342 | 440698 | 3, 4 |
| ch07 | 579231 | 582804 | 2, 4 |
| ch07 | 799618 | 802065 | 2, 3 |
| ch07 | 849139 | 850855 | 1, 2 |
| ch07 | 941031 | 943674 | 1, 2 |
| ch08 | 203881 | 209089 | 2, 4 |
| ch08 | 211544 | 218191 | 1, 3 |
| ch08 | 397790 | 398621 | 3, 4 |
| ch08 | 513025 | 518911 | 2, 3 |
| ch09 | 95341 | 97661 | 2, 4 |
| ch09 | 274412 | 274971 | 1, 3 |
| ch09 | 298781 | 303587 | 1, 3 |
| ch10 | 78221 | 81026 | 3, 4 |
| ch10 | 120287 | 121721 | 1, 2 |
| ch10 | 279698 | 282733 | 1, 4 |
| ch10 | 331929 | 334120 | 2, 4 |
| ch10 | 465014 | 466324 | 2, 4 |
| ch10 | 558307 | 560030 | 1, 3 |
| ch11 | 24444 | 24925 | 1, 2 |
| ch11 | 309395 | 310151 | 1, 2 |
| ch11 | 459289 | 460859 | 3, 4 |
| ch11 | 580805 | 585871 | 3, 4 |
| ch12 | 37551 | 46413 | 1, 4 |
| ch12 | 127005 | 128382 | 1, 3 |
| ch12 | 317990 | 325272 | 3, 4 |
| ch12 | 397606 | 400452 | 2, 3 |
| ch12 | 609793 | 616058 | 2, 3 |
| ch12 | 872179 | 875362 | 3, 4 |
| ch12 | 986364 | 986651 | 1, 3 |
| ch12 | 1054966 | 1058605 | 1, 3 |
| ch13 | 41051 | 43096 | 1, 4 |
| ch13 | 156894 | 161085 | 1, 4 |
| ch13 | 221398 | 223396 | 1, 3 |
| ch13 | 382193 | 382383 | 2, 4 |
| ch13 | 773436 | 774680 | 2, 4 |
| ch13 | 874295 | 876529 | 3, 4 |
| ch14 | 19742 | 23883 | 2, 3 |
| ch14 | 106213 | 107444 | 2, 3 |
| ch14 | 262514 | 263037 | 2, 3 |
| ch14 | 422748 | 431065 | 2, 4 |
| ch14 | 464235 | 467030 | 3, 4 |
| ch14 | 505442 | 507430 | 3, 4 |
| ch14 | 639289 | 644951 | 3, 4 |
| ch14 | 755402 | 757948 | 1, 2 |
| ch15 | 19470 | 27792 | 3, 4 |
| ch15 | 215175 | 222859 | 1, 3 |
| ch15 | 301320 | 303068 | 2, 4 |
| ch15 | 481862 | 483753 | 2, 4 |
| ch15 | 574822 | 577828 | 1, 4 |
| ch15 | 745307 | 747553 | 1, 4 |
| ch15 | 826033 | 827283 | 2, 4 |
| ch15 | 923564 | 926123 | 1, 3 |
| ch15 | 984989 | 988093 | 1, 4 |
| ch16 | 47416 | 51430 | 2, 3 |
| ch16 | 253089 | 256671 | 1, 2 |
| ch16 | 292698 | 299677 | 1, 3 |
| ch16 | 318071 | 325849 | 2, 3 |
| ch16 | 357944 | 360697 | 1, 4 |
| ch16 | 504268 | 507167 | 1, 3 |
| ch16 | 620612 | 625480 | 2, 4 |
| ch16 | 695784 | 700040 | 2, 3 |
| ch16 | 830978 | 832984 | 3, 4 |
| ch16 | 897732 | 902925 | 3, 4 |

Table S2. List of all 21 GCs for all 16 chromosomes

| Chromosome | Start position | End position | Converted product |
| --- | --- | --- | --- |
| ch02 | 256265 | 256939 | 4 |
| ch02 | 545432 | 546927 | 3 |
| ch03 | 63611 | 63611 | 2 |
| ch04 | 130712 | 132561 | 1 |
| ch04 | 263532 | 265026 | 3 |
| ch05 | 106481 | 107580 | 3 |
| ch06 | 76001 | 77338 | 1 |
| ch06 | 252258 | 252258 | 1 |
| ch07 | 124766 | 127355 | 1,3 |
| ch07 | 392320 | 392320 | 2 |
| ch07 | 668532 | 668532 | 2 |
| ch08 | 277105 | 278112 | 2 |
| ch10 | 110554 | 112235 | 1 |
| ch10 | 641747 | 642373 | 1 |
| ch11 | 235688 | 237470 | 2 |
| ch12 | 567959 | 568548 | 4 |
| ch12 | 899794 | 906336 | 4 |
| ch13 | 625678 | 626429 | 3 |
| ch13 | 852528 | 852528 | 1 |
| ch15 | 494572 | 495046 | 2 |
| ch16 | 694512 | 694512 | 4 |
